# A novel three-axis cylindrical hohlraum designed for inertial confinement fusion ignition

**DOI:** 10.1038/srep34636

**Published:** 2016-10-05

**Authors:** Longyu Kuang, Hang Li, Longfei Jing, Zhiwei Lin, Lu Zhang, Liling Li, Yongkun Ding, Shaoen Jiang, Jie Liu, Jian Zheng

**Affiliations:** 1 Research Center of Laser Fusion, China Academy of Engineering Physics, P.O. Box 919-986, Mianyang 621900, China; 2 CAS Key Laboratory of Basic Plasma Physics and Department of Modern Physics, University of Science and Technology of China, Hefei 230026, China; 3 Center of Fusion Energy Science and Technology, Beijing 100088, China; 4 Collaborative Innovation Center of IFSA, Shanghai Jiao Tong University, Shanghai 200240, China; 5 Institute of Applied Physics and Computational Mathematics, Beijing 100088, China

## Abstract

A novel ignition hohlraum for indirect-drive inertial confinement fusion is proposed, which is named three-axis cylindrical hohlraum (TACH). TACH is a kind of 6 laser entrance holes (LEHs) hohlraum, which is orthogonally jointed of three cylindrical hohlraums. Laser beams are injected through every entrance hole with the same incident angle of 55°. A view-factor simulation result shows that the time-varying drive asymmetry of TACH is less than 1.0% in the whole drive pulse period without any supplementary technology. Coupling efficiency of TACH is close to that of 6 LEHs spherical hohlraum with corresponding size. Its plasma-filling time is close to that of typical cylindrical ignition hohlraum. Its laser plasma interaction has as low backscattering as the outer cone of the cylindrical ignition hohlraum. Therefore, TACH combines most advantages of various hohlraums and has little predictable risk, providing an important competitive candidate for ignition hohlraum.

In indirect-drive Inertial Confinement Fusion (ICF), laser beams are injected into a high-Z hohlraum through laser entrance holes (LEHs) and are converted into X-ray radiation, then the radiation irradiates a low-Z capsule in the center of the hohlraum to bring the central fuel in the capsule to ignition conditions^1^,^2^,^3^,^4^,^5^,^6^. To achieve the ignition conditions, a convergence ratio of about 30 is necessary in the central hot spot ignition scheme^1^,^3^, so the radiation drive asymmetry should be less than 1%^1^, which is the key point for hohlraum design. Up to now, cylindrical hohlraum with 2 LEHs is the main choice and has been largely studied in the National Ignition Campaign (NIC)^7^. In order to achieve necessary time-varying symmetry in cylindrical hohlraums, multi-cone laser beams are used, and the P_2_ and P_4_ asymmetries are controlled by adjusting the power ratio between the inner and outer cones (beam phasing technology)^1^,^4^. However, the inner cone beams generate a considerable fraction of backscattering^7^, and the overlap of multiple cones causes crossed-beam energy transfer^8^,^9^,^10^. In addition, the plasma bubbles generated by outer cone affect the transfer of inner beams. These problems make the beam phasing a very complicated job. In addition, the beam phasing technology strictly depends on simulations, and the plasma of laser plasma interaction (LPI) region is non-local thermodynamic equilibrium, so it is difficult to be accurately calculated^11^. Besides cylindrical hohlraum, other hohlraums with different shapes have been proposed and investigated to improve the radiation environment inside the hohlraums, such as rugby hohlraum^12^,^13^,^14^, 4 or 6 LEHs spherical hohlraum^15^,^16^,^17^,^18^,^19^,^20^. However, rugby hohlraum has the similar problem of beam phasing as cylindrical hohlraum. For 4 LEHs spherical hohlraum, it is difficult to control asymmetry below 1.0% using single cone beams^16^,^18^. 6 LEHs spherical hohlraum has the natural superiority in radiation symmetry^18^, but experimental studies near ignition conditions are difficult and insufficiency on existing facilities.

## Results

### A novel three-axis cylindrical hohlraum

**Figure 1:**
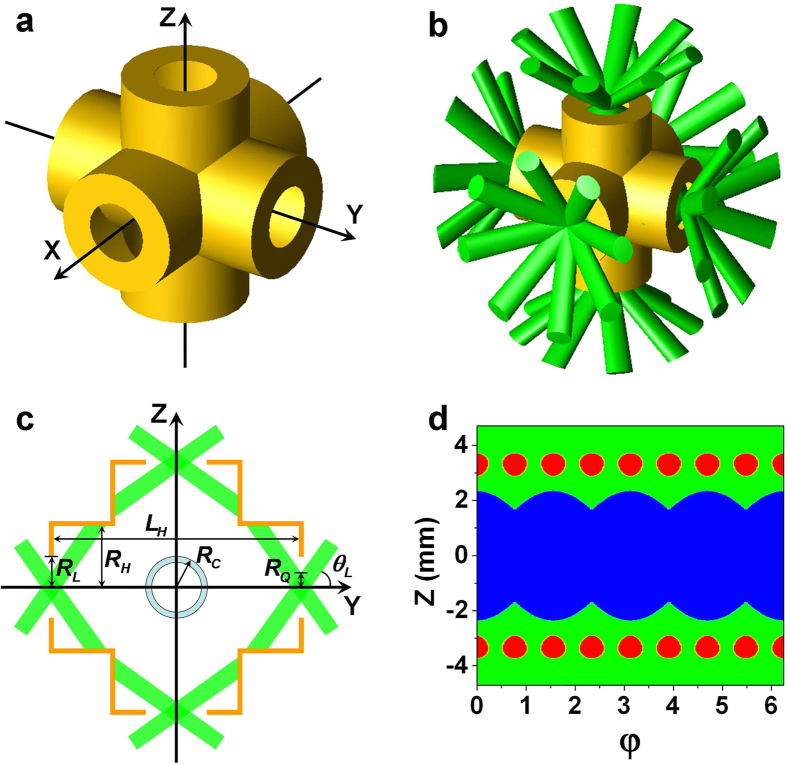
Schematics of TACH. (**a**) 3D structure of TACH, (**b**) Laser arrangement for TACH, (**c**) Schematic of TACH on the Y-Z plane, (**d**) Lateral expansion graph of the cylindrical hohlraum whose axis coincides with the Z axis. Red region indicates laser spots on inner wall, green region indicates X-ray reemission region on inner wall, and blue region indicates inane region incised by the other two cylindrical hohlraums.

Based on plentiful simulation studies of various hohlraums and existing experimental results on cylindrical hohlraum, a novel hohlraum named three-axis cylindrical hohlraum (TACH) is proposed, which is orthogonally jointed of three cylindrical hohlraums, as shown in Fig. 1. The axes of the three cylindrical hohlraums coincide with the X, Y, Z axis of a rectangular coordinate system respectively. TACH is a kind of 6 LEHs hohlraum. 8 laser quads arranged in one cone are injected through each LEH. The time-varying symmetry, coupling efficiency and plasma filling of TACH are studied in this article.

### Time-varying symmetry

**Figure 2:**
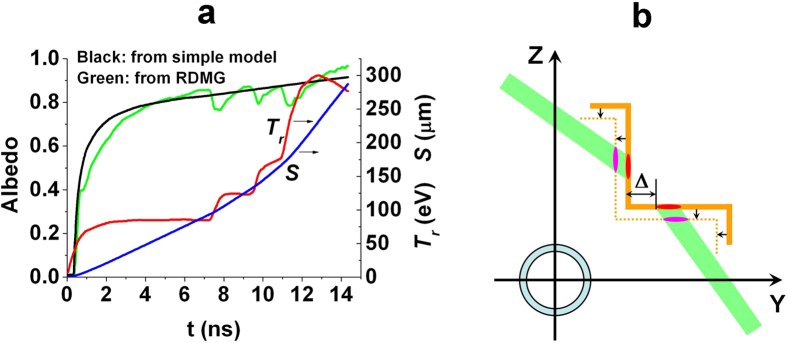
Albedos evolution and hohlraum wall motion. (**a**) Evolution of the albedo from simple model (black), the albedo from RDMG simulation (green), the radiation temperature *T*_*r*_ (red) and displacement *s* of hohlraum wall (blue). (**b**) Schematic of hohlraum wall motion and LEHs closure. Solid golden line indicates initial position of the hohlraum wall, and dashed golden line indicates final position. Green strips indicate laser beams, whose overlapping regions with hohlraum wall roughly indicate the locations of laser spots. The loop near the original point indicates the capsule region. Δ indicates the minimum distance between the initial edge of the laser spots and the initial inner wall of adjacent cylindrical hohlraum.

Due to the single-cone design for TACH, it is hard to control the time-varying radiation drive asymmetry by beam phasing technology, so it is crucial to study the time-varying symmetry of TACH. The symmetry can be studied by use of a view-factor model^21^,^22^,^23^. In order to simplify the analysis, the radiation intensity of the laser spots and that of the reemission wall are assumed to be uniformly distributed respectively. *F*_*S*_ and *F*_*W*_ are used to denote the radiation intensities of the two kinds of regions. *T*_*r*_ shown in Fig. 2(a) is chosen as the ignition radiation temperature pulse inside the hohlraum^4^,^24^. *α*_*W*_ and *α*_*C*_ are defined as the albedo of hohlraum wall and that of capsule respectively. *A*_*W*_, *A*_*S*_, *A*_*C*_, and *A*_*L*_ are used to denote the areas of the hohlraum wall, laser spots, capsule surface and LEH respectively. Among them, *A*_*S*_ is a part of *A*_*W*_. According to the power balance equation^25^,


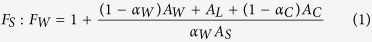


A simple analytical model is used to calculate the time-varying albedo of the hohlraum wall. During 0 ns~7 ns, the albedo is calculated by the scaling law 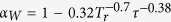 2 and *α*_*W*_ is taken as 0.01 if *α*_*W*_ < 0.01. Then it increases linearly after 7 ns and reaches 0.9 at 13 ns^22^,^26^ as shown in Fig. 2(a). The albedo calculated by the simple model (black) is close to the simulation result from the 1D radiation hydrodynamic code of multi-groups (RDMG)^14^ (green), so it is reasonable to be used in our analysis.

With the ablation of laser and x-ray, the hohlraum walls move inward. Study shows that the inward wall movement is dominated by reradiated x-ray ablation, not by laser ablation^2^. Considering the quasi-uniform radiation environment inside the hohlraum, it can be assumed that the whole hohlraum walls including the LEH move inward at the same speed. Figure 2(b) is the schematic of the hohlraum wall movement and the LEHs closure. According to the theory of radiation ablation^3^, the speed (*v*) of hohlraum wall movement can be assumed to be proportional to the sound speed *c*_*s*_ of the high-Z ablated plasma. For Au hohlraum wall, the sound speed is 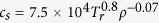 1, where *T*_*r*_ is the radiation temperature in eV, *ρ* is the density of ablated plasma in g/cm^3^. Ignoring the weak relationship between *c*_*s*_ and *ρ*, it is simplified down to 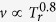. It is assumed the distance of inward wall motion is 275 μm at 14 ns^19^,^22^,^27^. According to the *T*_*r*_ pulse shown in Fig. 2(a), the speed of hohlraum wall can be written as 
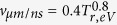
. The displacement *s* of hohlraum wall can be calculated by integrating the velocity over time as shown in Fig. 2(a).

As shown in Fig. 1(c), the laser quads are injected into the hohlraum with incident angle of *θ*_*L*_, and the profile of laser spot on the LEH plane is set as round with radius of *R*_*Q*_. *R*_*H*_, *R*_*L*_ and *R*_*C*_ indicate the radius of cylindrical hohlraum, LEH and capsule respectively. Δ indicates the minimum distance between the initial edge of the laser spots and the initial inner wall of adjacent cylindrical hohlraum as is shown in Fig. 2(b). So the length of the cylindrical hohlraum can be written as,


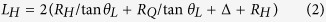


In the calculation model, *R*_*Q*_ = 0.6 mm, *α*_*C*_ = 0.3^20^,^28^,^29^ and *R*_*C*_ = 1.18 mm^4^,^24^ are taken, which are all independent of time, and the initial *R*_*L*_ is taken as 1.3 mm. By choosing the appropriate Δ to set the initial positions of laser spots and considering the time-varying albedo and displacement of hohlraum wall, the time-varying radiation asymmetry on capsule 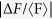 can be calculated, where F is the radiation intensity on capsule, 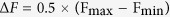 and 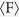 is the average value of flux F on capsule^18^.

**Figure 3:**
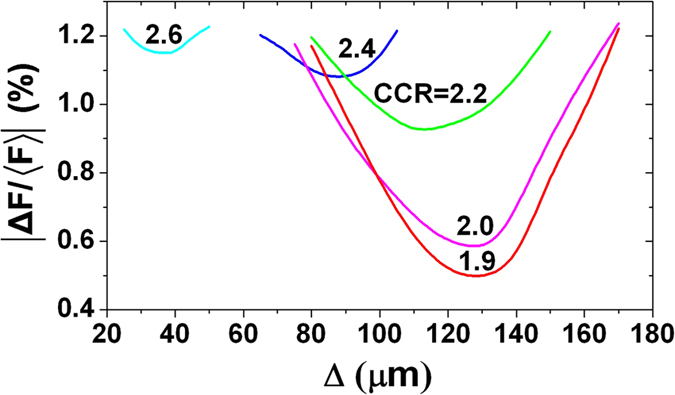
Symmetries vary with Δ for hohlraums with different CCR near the optimum Δ. A smaller CCR corresponds to a better initial symmetry and a larger optimum Δ.

In the calculation, *R*_*H*_/*R*_*C*_ is defined as the case-to-capsule ratio (CCR). The radiation drive symmetries of the hohlraums with CCRs = 1.9, 2.0, 2.2, 2.4 and 2.6 are investigated with the laser incident angle of 55°. In the early stage *α*_*W*_ << 1, and it can be calculated from Eq. (1) that the ratio of radiation intensity of laser spots to that of reemission wall *F*_*s*_/*F*_*W*_ >> 1. So the early symmetry is mainly determined by the initial position of the laser spots. In order to avoid the case that the laser beams from one cylindrical hohlraum enter another one, Δ ≥ 0 is taken as shown in Fig. 2(b). Calculations indicate that there is an optimum Δ in order to obtain the best initial symmetry for TACHs with different dimensions. Symmetry variation with Δ near the optimum Δ is illustrated in Fig. 3, and a smaller CCR corresponds to a better initial symmetry and a larger optimum Δ.

**Figure 4:**
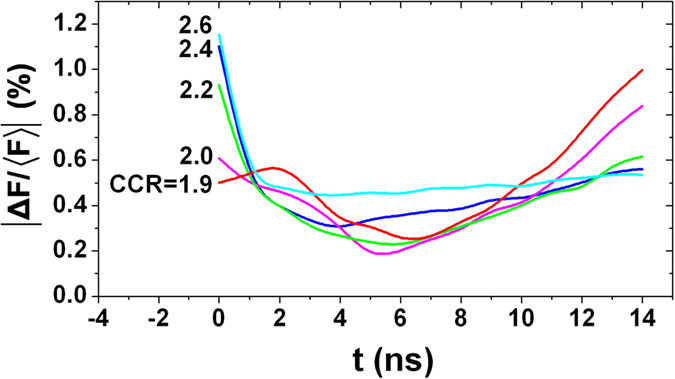
Time-varying symmetries of TACHs with different CCR (*θ*_*L*_ = 55°).

By setting the laser spots at the optimized initial position, the time-varying radiation drive symmetries of TACHs are calculated by use of the view-factor model mentioned above. The time-varying symmetries of TACHs with different CCR are shown in Fig. 4, which indicate that all the hohlraums with CCR from 1.9 to 2.6 have a good time-varying symmetry during the whole period of the laser pulse. The initial asymmetry increases with the CCR of the hohlraums. Nevertheless, even for the hohlraum with the greatest CCR of 2.6, the initial 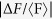 is below 1.2%, and falls rapidly to below 1.0% after 300 ps with the heating of the wall and the rising of the albedo. In the cases of CCR ≤ 2.2, the initial 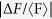 is less than 1.0%, meeting the need of the initial symmetry for ignition. During the period between 1 ns and 11 ns, all the 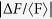 keep below 0.6%, the minimum value is even less than 0.2%. During the main pulse of the radiation (11 ns~14 ns), the asymmetry increases nearly linearly with time, a smaller CCR corresponds to a quicker increase of the asymmetry. At the end of the radiation pulse (t = 14 ns), the smaller asymmetry is achieved for hohlraums with larger CCR, which is just opposite from the early situations, and all of the asymmetry can be controlled below 1.0% at this time. After 1 ns, the asymmetry changes slower for the hohlraum with a larger CCR, especially for the hohlraum with CCR = 2.6 whose 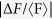 keeps below 0.6% and almost independent of time from 1 ns to 14 ns.

In general, a smaller CCR corresponds to a better early symmetry while a larger CCR corresponds to a better final symmetry. Take the implosion of the capsule into consideration, a small initial CCR becomes larger and larger during the laser pulse, which is beneficial to improve the final symmetry of the TACH. So the TACHs with CCR = 2.0~2.2 are selected as the optimum hohlraum.

**Figure 5:**
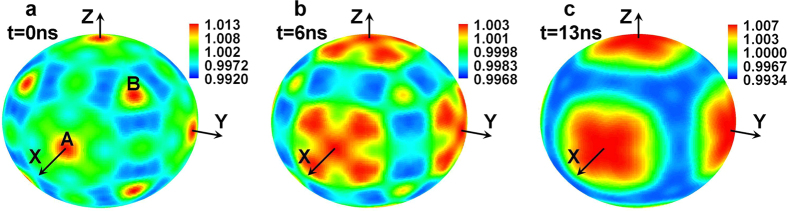
Distribution of normalized drive flux on the capsule at the moment of t = 0 ns, 6 ns and 13 ns respectively (CCR = 2.2, *θ*_*L*_ = 55°).

Similar to the case of 6 LEHs spherical hohlraum^18^, as shown in the Fig. 5(a), there are two special types of points on the capsule. The normal directions of the first type points are parallel or antiparallel to the direction of X, Y or Z axis, and are facing the corresponding LEHs. One of such six points is marked with A in Fig. 5(a). For another type of points, the normal direction of which has an equal angle with X, Y and Z axis, adds up to 8, and one of such points is marked with B in Fig. 5(a). Calculations indicate that the initial symmetry on the capsule is optimal if the drive flux of A is equal to that of B. Then the relative flux of regions around point A become more and more intense than that of regions around point B due to the movement of the laser spots towards the LEHs with time. At the end of the laser pulse, the radiation flux into the points facing the LEHs are more intense than other points of the capsule.

**Figure 6:**
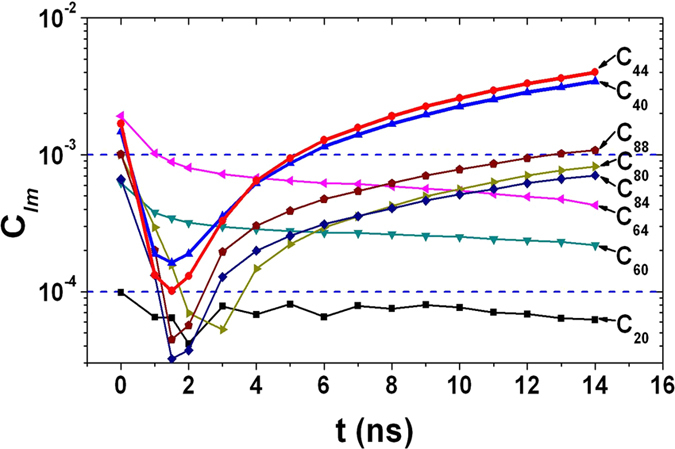
The time-varying spherical-harmonic expansion of drive flux on capsule (CCR = 2.2, *θ*_*L*_ = 55°).

Using spherical harmonic function *Y*_*lm*_(*θ*, *φ*), the drive flux on capsule can be expanded as 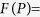

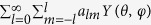
, where *F*(*P*) is the drive flux at point *P*(*θ*, *φ*) on capsule, and *a*_*lm*_ is spherical harmonic decomposition. Define *C*_*l0*_ = |*a*_*l0*_|/*a*_*00*_ and *C*_*lm*_ = |2*a*_*lm*_|/*a*_*00*_ for *m* > 0, and the time-varying *C*_*lm*_ is shown in Fig. 6. During the whole drive period, C_20_ can be suppressed below 0.01%, which can be neglected. For the modes with *l* ≥ 6, the maximum value of them is less than 0.2%, and the time-varying values of them can be controlled below 0.1% at most time. After 5 ns, C_40_ and C_44_ dominate the capsule flux asymmetry, both of which increase monotonously from 0.1% to about 0.4%. As shown in Fig. 5, this is mainly because the relative flux of regions around point A becomes more and more intense due to the movement of the laser spots towards the LEHs with time, which generates the distribution characteristic of C_40_ and C_44_. Nevertheless, 0.4% asymmetry of C_40_ and C_44_ meets the need of drive symmetry for ignition.

TACH has the similar symmetrical characteristic with regular hexahedron, which is the underlying physics for TACH to achieve high drive symmetry. As the analysis of spherical-harmonic expansion shows, the symmetrical arrangement of TACH can suppress C_20_ to a very low level and the asymmetry is dominated by C_40_ and C_44_, which is similar to 6 LEHs spherical hohlraum.

**Figure 7:**
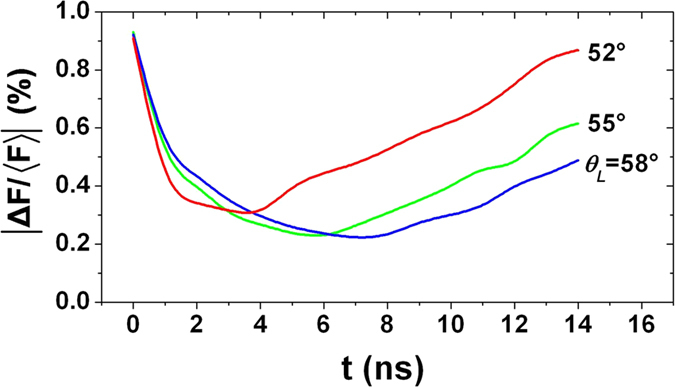
Time-varying symmetries vary with the incident angle of laser beams.

The time-varying symmetries varying with the incident angle of laser beams were also investigated under the condition of CCR = 2.2, as illustrated in Fig. 7. The results show that the initial symmetry has weak relationship with the incident angle. However, this situation changes after 6 ns. The time-varying symmetry becomes better with larger incident angle. The reason is that the distance of laser spots moving along the hohlraum axis is much smaller at a greater incident angle due to the inward movement of the hohlraum wall. Considering the trade-offs between time-varying symmetry and the laser injection convenience, 55° is chosen as the optimum incident angle of the laser beams.

Based on the above analysis, the optimum size of TACH is CCR = 2.0~2.2 and the optimum incident angle of laser is 55°.

### Coupling efficiency

Define “coupling efficiency” as the ratio of absorbed energy *E*_*C*_ by capsule to the incident laser energy *E*_*L*_, which is given by


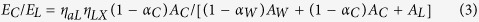


where *η*_*aL*_ is the fraction of laser absorbed, and *η*_*LX*_ is the laser to x-ray conversion efficiency.

**Figure 8:**
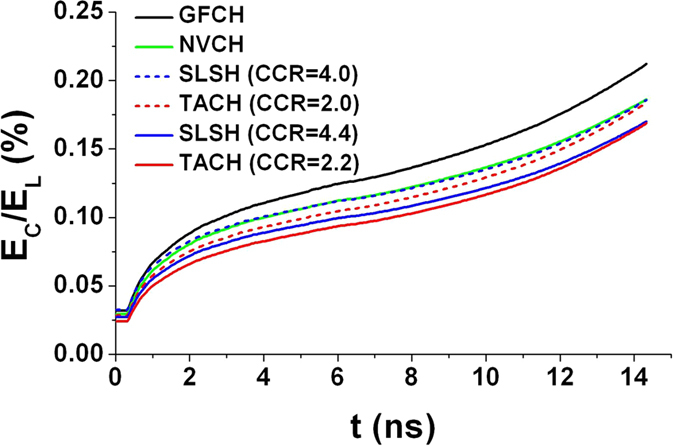
Time-varying coupling efficiency comparison of GFCH (*R*_*H*_ = 2.95 mm, *L* = 10.6 mm, LEH ø3.1 mm), NVCH (*R*_*H*_ = 3.36 mm, *L* = 11.26 mm, LEH ø3.9 mm), SLSH (CCR = 4.0, or CCR = 4.4, LEH ø2.6 mm) and TACH (CCR = 2.0, or CCR = 2.2, LEH ø2.6 mm). Capsules with same *R*_*C*_ = 1.18 mm are adopted.

The coupling efficiencies of several hohlraums are compared, including gas-filled cylindrical hohlraum (GFCH), near-vacuum cylindrical hohlraum (NVCH), six LEHs spherical hohlraum (SLSH) and TACH (CCR = 2.0, 2.2). For the GFCH, *η*_*aL*_ is about 0.85 due to the strong backscatter from the inner cone beams^7^. For the NVCH, *η*_*aL*_ is about 0.96^30^. For SLSH and TACH, *η*_*aL*_ is about 0.96, because the properties of LPI of SLSH and TACH are close to those of the outer cone of GFCH with a low level of backscattering. *η*_*LX*_ is about 0.8 for all kinds of hohlraums^31^. Time-varying coupling efficiency comparison of GFCH (*R*_*H*_ = 2.95 mm, *L* = 10.6 mm, LEH ø3.1 mm)^4^,^24^, NVCH (*R*_*H*_ = 3.36 mm, *L* = 11.26 mm, LEH ø3.9 mm)^32^, SLSH (CCR = 4.0, 4.4, LEH ø2.6 mm) and TACH (CCR = 2.0, 2.2, LEH ø2.6 mm) are calculated and shown in Fig. 8. The calculation results show that the time-varying coupling efficiency of TACH with CCR0 is very close to that of SLSH with 2 × CCR0. The coupling efficiencies of TACH (CCR = 2.0), SLSH (CCR = 4.0) and NVCH are close to each other, which is about 13% lower than that of GFCH. The coupling efficiency of TACH (CCR = 2.2) is similar with SLSH (CCR = 4.4), which is about 20% lower than that of GFCH. Nevertheless, it is worthwhile to spend 13%~20% more laser energy for a higher symmetry during the whole period of implosion of capsule. Furthermore, the coupling efficiency of TACH can be increased by above 10% using LEH shields^20^. Compared with the arrangement of multi-cone lasers for cylindrical hohlraum, the arrangement of single-cone lasers with large angle can supply sufficient space to place LEH shields.

### Plasma filling

The plasma ablated from the hohlraum wall will fill the volume inside the hohlraum, which would affect the injection of laser and limit the performance of hohlraum. The plasma filling time of TACH and cylindrical hohlraum are compared to evaluate the plasma filling problem of TACH. Filling model in ref. 33 is only used in vacuum cylindrical hohlraums, which is extended to gas-filled hohlraums with arbitrary shape in this article. The improved model is shown in Eq. (4), whose detail is shown in the Methods section.


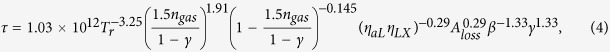


where *τ* is used to denote the filling time in ns, and *n*_*gas*_ is the initial electron density of gas in *n*_*c*_. Helium is chosen as the filled gas. Filling ratio *γ* = *V*_*fill-wall*_/*V*_*H*_, where *V*_*fill-wall*_ is the filled volume of radiation-ablated plasma and *V*_*H*_ is the hohlraum volume. *β* = *A*_*W*_/*V*_*H*_. Equivalent energy loss area *A*_*loss*_ = (1 *−* *α*_*W*_)*A*_*W*_ + (1 − *α*_*C*_)*A*_*C*_ + *A*_*L*_.

For a radiation temperature *T*_*r*_ and an initial filling gas density *n*_*gas*_, the filling model can be used to calculate the filling time it takes for the hohlraum to fill to a filling ratio *γ*. It can be seen from Eq. (4) that a higher density of initial filling gas or a larger filling ratio corresponds to a longer filling time. For a certain *T*_*r*_, *n*_*gas*_, *γ*, *η*_*aL*_ and *η*_*LX*_, plasma filling time


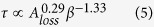


**Figure 9:**
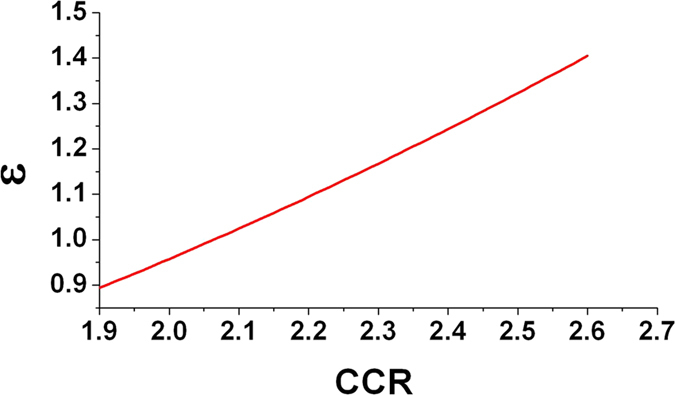
Ratio of filling time of TACH (Δ = 100 μm, LEH ø2.6 mm) to filling time of cylindrical hohlraum (*R*_*H*_ = 2.95 mm, *L* = 10.6 mm, LEH ø3.1 mm) varying with CCR.

Define ε as the ratio of the filling time of TACH to the filling time of cylindrical hohlraum. Take *α*_*W*_ = 0.8, *α*_*C*_ = 0.3, *R*_*C*_ = 1.18 mm for two kinds of hohlraum. For TACH (Δ = 100 μm, LEH ø2.6 mm) and cylindrical hohlraum (*R*_*H*_ = 2.95 mm, *L* = 10.6 mm, LEH ø3.1 mm), ε can be calculated from Eq. (5). Figure 9 shows the ε varying with CCR of TACH. For TACHs with CCR between 2.0 and 2.2, ε is between 0.96 and 1.1, so the filling time of TACH is close to cylindrical hohlraum.

## Discussion

From another point of view, TACH is composed of six half cylindrical hohlraums (HCHs). For laser injection, the six HCHs can be decoupled from each other, which brings great convenience for laser arrangement. Furthermore, the lasers and plasma condition in each HCH are mainly cylindrical symmetry, so these can be studied approximately by a cylindrical 2D radiation hydrodynamic model. Single cone lasers are injected into each HCH with large incident angle, which is similar to the outer cone of the ignition cylindrical hohlraum in NIC. Therefore, it is reasonable to predict that the backscattering of TACH is as slight as that of the outer cone of the ignition cylindrical hohlraum. In addition, single-cone injection avoids several other LPI problems of multi-cone cylindrical hohlraums, such as crossed-beam energy transfer between two laser cones, and blocking of the transfer of inner-cone laser by high-Z plasma bubbles ablated by outer-cone lasers. Moreover, single-cone injection greatly simplifies the symmetry tuning. To optimize the symmetry of a certain TACH, it is only need to adjust the initial position of laser spot and control the power balance of laser. The parameters of TACH can be optimized to control the time-varying asymmetry below 1.0% during the whole drive pulse. The filling time of TACH is close to that of typical ignition cylindrical hohlraum in NIC. Although the coupling efficiency of TACH is about 13%~20% lower than that of ignition cylindrical hohlraum, it is worthwhile to spend 13%~20% more laser energy for the superiorities of TACH as discussed above. Therefore, TACH combines most advantages of various hohlraums and has little predictable risk, providing an important potential way for ignition hohlraum design in ICF.

## Methods

### The time-varying symmetry calculation by use of view factor model

For a given time, the size and the albedo of TACH can be calculated by the corresponding models as described above. Then the hohlraum wall is divided into zones, and the size of each zone is controlled below 10 μm to ensure adequate resolution. The positions and areas of laser spots are determined by the intersection between the laser beams and the hohlraum wall. Based on this, the ratio of radiation intensity of laser spots to that of re-radiated wall can be calculated by Eq. (1). δ is used to denote the ratio, so F_W_ and F_S_ can be set as 1 and δ respectively, which is normalized by F_W_. For a zone near the edge of the laser spots, maybe only a part of the zone is in the spot, the radiation intensity of the zone is calculated by area weighting. For example, S_0_, S_1_ and S_2_ denote the total area of this zone, the area of this zone inside and outside the laser spot respectively, so the intensity of this zone can be calculated by (δS_1_ + S_2_)/S_0_. After setting the normalized distribution of radiation intensity, the radiation symmetry of capsule can be calculated by the view factor model. In the calculation, the drive contribution from each cylindrical hohlraum can be calculated independently, and only the self-block of capsule needs to be considered.

### The extending of filling model

*V*_*H*_ is defined as the hohlraum volume. *V*_*fill-wall*_ and *V*_*fill-gas*_ are the filled volume of radiation-ablated plasma and that of gas plasma respectively. Ignoring the volume of laser channels in Au plasma, *V*_*H*_ = *V*_*fill-wall*_ + *V*_*fill-gas*_. Define *β* = *A*_*W*_/*V*_*H*_ and filling ratio *γ* = *V*_*fill-wall*_/*V*_*H*_. *n*_*gas*_ is used to denote the initial electron density of gas. Helium is chosen as the filled gas. *n*_*e−c*_, *n*_*e−w*_ and *n*_*e−g*_ are defined as the electron density of Au plasma in laser channel, radiation-ablated plasma and gas plasma respectively. *T*_*e−c*_ is defined as the electron temperature of Au plasma in the laser channel. Z_*c*_ is the ionization degree of Au plasma in laser channel and Z_*w*_ is the ionization degree of radiation-ablated plasma. Laser wavelength is 351 nm. Equivalent energy loss area *A*_*loss*_ = (1 *−* *α*_*W*_)*A*_*W*_ + (1 *−* *α*_*C*_)*A*_*C*_ + *A*_*L*_.

Unit selection: laser power *P*_0_ uses TW, radiation temperature *T*_*r*_ uses eV, electron temperature *T*_*e−c*_ uses keV, length uses cm, *n*_*gas*_, *n*_*e−c*_, *n*_*e−w*_ and *n*_*e−g*_ use *n*_*c*_, and filling time *τ* uses ns.

According to the power balance of hohlraum,


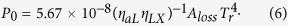


According to the power balance of laser channel^33^,


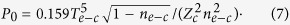


According to the pressure balance between laser channel plasma and radiation-ablated plasma,


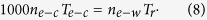


The ion and electron temperatures of the gas plasma are supposed equal to the electron temperature of Au plasma in laser channel. According to the pressure balance between gas plasma and radiation-ablate plasma,


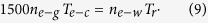


Using filling ratio γ, *n*_*e−g*_ can be calculated by


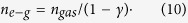


Using Eqs. (128) and (129) in ref. 1 to calculate radiation-ablated mass rate, and ablated plasma from the hohlraum wall *A*_*W*_ is supposed to uniformly fill in the volume of *V*_*fill-wall*_.


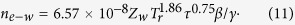


The ionization degree of radiation-ablated plasma calculated from Eq. (187) in ref. 1


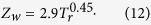


The ionization degree of Au plasma in the laser channel^33^


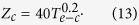


Combining Eqs. (6, 7, 8, 9, 10, 11, 12, 13) and solving for τ, the improved filling model can be got as shown in Eq. (4).

## Additional Information

**How to cite this article**: Kuang, L. *et al.* A novel three-axis cylindrical hohlraum designed for inertial confinement fusion ignition. *Sci. Rep.* **6**, 34636; doi: 10.1038/srep34636 (2016).
